# Rectal lymph node metastasis in recurrent ovarian carcinoma: essential role of ^18^F-FDG PET/CT in treatment planning

**DOI:** 10.1186/1477-7819-11-184

**Published:** 2013-08-12

**Authors:** Koji Kumagai, Terue Okamura, Masao Toyoda, Hideto Senzaki, Chihiro Watanabe, Masahide Ohmichi

**Affiliations:** 1 Department of Obstetrics and Gynecology, Takatsuki Red Cross Hospital, Takatsuki-city, Osaka 569-1096, Japan; 2 Department of Pathology, Takatsuki Red Cross Hospital, Takatsuki-city, Osaka 569-1096, Japan; 3 PET Center, Osaka Saiseikai Nakatsu Hospital, Osaka-city, Osaka 530-0012, Japan; 4 Department of Surgery, Osaka Saiseikai Nakatsu Hospital, Osaka-city, Osaka 530-0012, Japan; 5 Department of Pathology, Osaka Saiseikai Nakatsu Hospital, Osaka-city, Osaka 530-0012, Japan; 6 Department of Obstetrics and Gynecology, Osaka Medical College, Takatsuki-city, Osaka 569-1096, Japan; 7 Department of Gynecology, Osaka Railway Hospital, 2-22, Matsuzakicho 1-chome, Abeno-ku, Osaka-city, Osaka 545-0053, Japan

**Keywords:** Recurrent ovarian carcinoma, Mesorectal lymph node, Pararectal lymph node, ^18^F-FDG PET/CT, Treatment planning

## Abstract

Although uncommon, ovarian cancer cells may spread to the rectal lymph nodes. However, few reports have described how to detect and treat such metastases. We report a case of a 59-year-old woman with mesorectal and pararectal lymph node metastases in recurrent ovarian carcinoma, detected conclusively using ^18^F-fluorodeoxyglucose (^18^F-FDG) positron emission tomography/computed tomography (PET/CT), and treated by low anterior resection with total mesorectal excision aiming for macroscopic complete resection. The treatment goals for the patient were gradually changed from curative to palliative chemotherapy; she survived for 45 months without rectal obstruction after secondary debulking surgery, and was followed up until autopsy. Thus, ^18^F-FDG PET/CT may be valuable for detecting rectal lymph node metastasis and can play an essential role in planning treatment for recurrent ovarian carcinoma.

## Background

Ovarian carcinoma is the most lethal gynecological malignancy, mainly because it extensively metastasizes to various sites through direct invasion, peritoneal dissemination, and lymphatic metastasis [1,2]. Ovarian cancer cells frequently spread to regional lymph nodes such as the iliac and para-aortic nodes [3]. Uncommonly, these cells may also spread to the rectal [4-7], inguinal [8], and intramammary [9] lymph nodes. However, few reports have described how to detect and treat such metastases [10]. Here, we report a case of a 59-year-old woman with mesorectal and pararectal lymph node metastases in recurrent ovarian carcinoma, who survived for 45 months after secondary debulking surgery (SDS) and was followed up until autopsy. We describe the essential role of ^18^F-fluorodeoxyglucose (^18^F-FDG) positron emission tomography/computed tomography (PET/CT) in treatment planning.

## Case presentation

**Figure 1:**
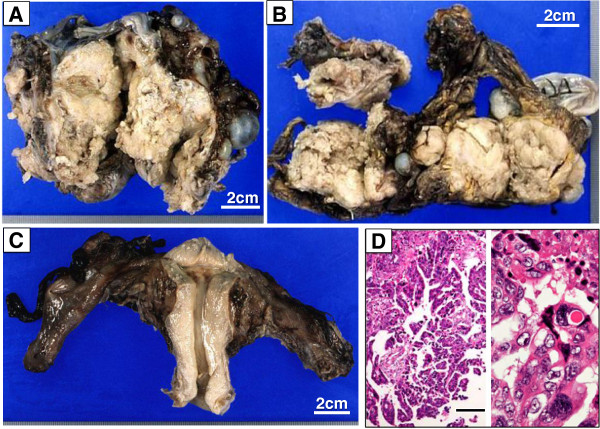
**Formalin-fixed specimens resected at primary surgery. (A)** Left ovarian tumor; **(B)** omentum with metastatic tumor; **(C)** uterus, right adnexa, and left fallopian tube; **(D)** microscopic view of the left ovarian tumor, showing serous papillary adenocarcinoma (hematoxylin and eosin stain, bar 100 μm).

**Figure 2:**
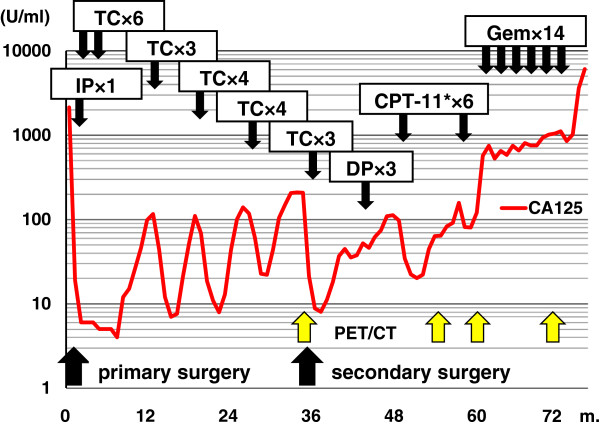
**Changes in cancer antigen 125 (CA125) levels during treatment.** The graph shows the treatments and the number of chemotherapeutic cycles. We used CPT-11 combined with cisplatin for three cycles, followed by three cycles of CPT-11 alone; ^18^F-fluorodeoxyglucose positron emission tomography/computed tomography was conducted four times (yellow arrows). CPT-11, camptothecin 11 (also known as irinotecan); DP, docetaxel and cisplatin; Gem, gemcitabine; IP, intraperitoneal chemotherapy with cisplatin; PET/CT, positron emission tomography/computed tomography; TC, paclitaxel and carboplatin.

A 59-year-old para 2 menopausal woman presented with a 15 cm × 15 cm pelvic tumor. Her mother had a history of breast cancer. The patient’s serum level of cancer antigen 125 (CA125) was elevated to 1,615 U/ml (normal value, <35 U/ml). Laparotomy showed that the tumor originated in the left ovary and was tightly attached to both the uterus and the rectum. Ascitic cytology (<100 ml) results were positive for adenocarcinoma. Consequently, the patient underwent total hysterectomy, bilateral salpingo-oophorectomy, and partial omentectomy (Figure 1). The maximum diameter of the remaining tumors was less than 1 cm. The operating time and blood loss were 265 min and 3,360 ml, respectively. The histological diagnosis was stage IIIc ovarian serous papillary adenocarcinoma, based on the 1994 International Federation of Gynecology and Obstetrics classification. Subsequently, she received intraperitoneal chemotherapy followed by adjuvant chemotherapy. Whenever her serum CA125 level exceeded 100 U/ml, she received repeated cycles of chemotherapy (Figure 2).

**Figure 3:**
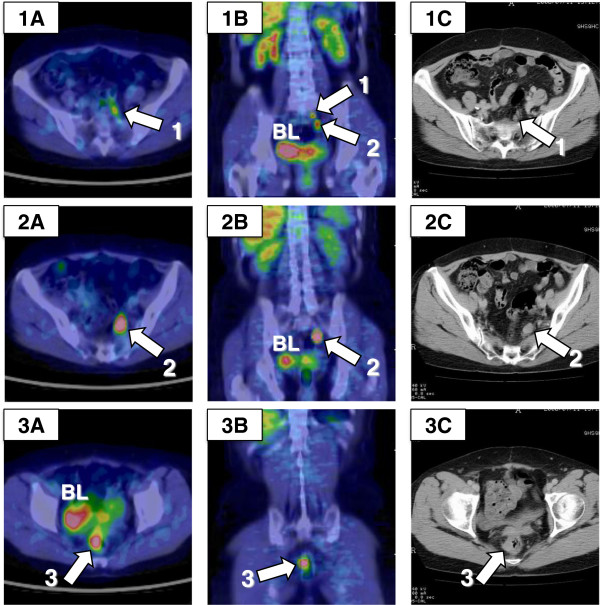
^**18**^**F-fluorodeoxyglucose positron emission tomography/computed tomography (**^**18**^**F-FDG PET/CT) images obtained 34 months after primary surgery. (A)** Axial and **(B)** coronal PET/CT images show three areas (1 to 3) of increased ^18^F-FDG uptake adjacent to the rectum. Maximum standardized uptake values (SUV_max_) at 60 and 120 min were **(1A)** 3.5 and 4.5, **(2A)** 9.4 and 13.7, and **(3A)** 6.4 and 7.6, respectively. **(C)** Axial contrast-enhanced CT scans were performed 2 weeks earlier, the results of which were equivocal for one mesorectal **(1C)** and one pararectal **(3C)** lymph node. BL, bladder.

**Figure 4:**
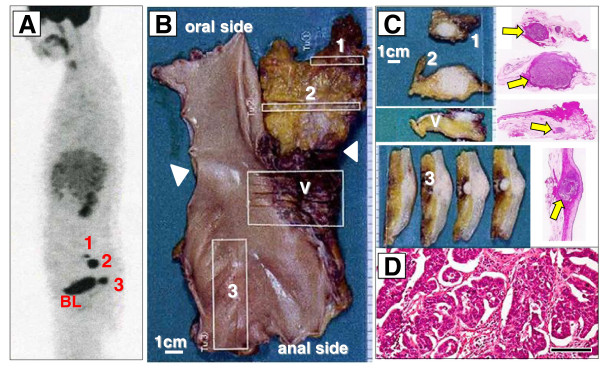
**Formalin-fixed specimens obtained at the time of the secondary debulking surgery. (A)** Intense uptake areas (1 to 3) in the ^18^F-fluorodeoxyglucose positron emission tomography (^18^F-FDG PET) maximum intensity projection image. **(B)** Specimen from the low anterior *en bloc* resection of the rectum; the triangles show the level of peritoneal reflex. **(C)** Sections 1 and 2 include mesorectal lymph nodes; section V (vagina) includes the posterior vaginal wall and a paravaginal lymph node; section 3 includes a pararectal lymph node; the arrows indicate loupe views of the swollen lymph nodes. **(D)** Microscopic view of section 3 (hematoxylin and eosin stain, bar 100 μm).

Thirty-four months after surgery, ^18^F-FDG PET/CT revealed increased ^18^F-FDG uptake in two mesorectal lymph nodes (12 and 26 mm) and one pararectal lymph node (18 mm) (Figure 3). The results of contrast-enhanced CT scans obtained 2 weeks earlier were equivocal for one mesorectal (12 mm) and one pararectal (18 mm) lymph node (Figure 3: 1A, 1C). The patient underwent SDS aimed at removing the three tumors; this surgery involved low anterior resection of the rectum with total mesorectal excision, along with resection of the vaginal cuff. On careful inspection of the abdominal cavity, no macroscopic peritoneal dissemination was identified. The surgery accomplished macroscopic complete resection. Histopathological analysis of the surgical specimens showed that the tumors were two mesorectal and one pararectal lymph node metastases; one paravaginal lymph node (5 mm) had not previously been detected by ^18^F-FDG PET/CT (Figure 4). The histology of all four of the lymph nodes was serous papillary adenocarcinoma, consistent with ovarian carcinoma (Figure 4D).

**Figure 5:**
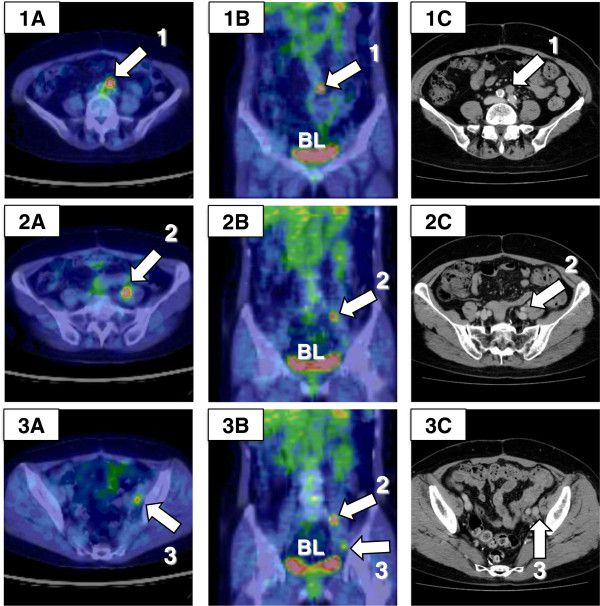
^**18**^**F-fluorodeoxyglucose positron emission tomography/computed tomography (**^**18**^**F-FDG PET/CT) images obtained 24 months after secondary debulking surgery. (A)** Axial and **(B)** coronal PET/CT images show three areas of increased ^18^F-FDG uptake (1 to 3) in the pelvic area. Maximum standardized uptake values (SUV_max_) at 60 and 120 min were **(1A)** 6.1 and 7.8, **(2A)** 3.9 and 6.2, and **(3A)** 2.7 and 4.1, respectively. **(C)** Axial contrast-enhanced CT scans performed 1 week earlier. BL, bladder.

**Figure 6:**
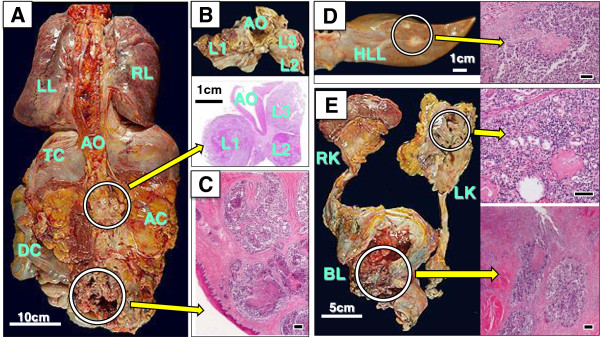
**Appearance at autopsy. (A)** Rear view of the internal organs. **(B)** Three para-aortic lymph node metastases (L1 to L3). **(C)** Vaginal wall involvement. **(D)** Two metastatic nodules on the liver surface. **(E)** Left kidney (LK) and bladder (BL) muscle involvement; RK, right kidney. Circles and arrows indicate loupe/microscopic views of the specimens (hematoxylin and eosin stain, bars 100 μm). AC, ascending colon; AO, aorta; BL, bladder; DC, descending colon; LK, left kidney; LL, left lung; HLL, hepatic left lobe; RK, right kidney; RL, right lung; TC, transverse colon.

^18^F-FDG PET/CT was performed at 19 (not shown), 24 (Figure 5), and 36 (not shown) months after SDS, and ^18^F-FDG uptake in the pelvic lymph nodes was observed. The patient received palliative chemotherapy until interstitial lung disease occurred. Forty-five months after SDS, the recurrent tumor invaded the vaginal wall and the patient died from vaginal hemorrhage, but without rectal obstruction throughout the clinical course. At autopsy, multiple metastases of the cancer were found, involving the bladder muscle, vaginal wall, pelvic wall, left kidney, pelvic and para-aortic lymph nodes, and liver surface (Figure 6).

Our imaging protocol required patients to fast for more than 5 h before intravenous injection of ^18^F-FDG (2.6 MBq/kg). After administration of ^18^F-FDG, an early emission scan (at 60 min) and delayed emission scan (at 120 min) were obtained in three-dimensional mode from the orbit to the upper thigh. All scans were performed with a PET/CT scanner (SET-3000BCT/L; Shimadzu, Kyoto, Japan), and transmission scans were simultaneously obtained using an external source (740 MBq ^137^Cs). After completion of the delayed PET scan, CT with a single-helical scanner was performed to reconstruct PET/CT images for visual interpretation and tumor volume measurements.

## Discussion

Here, we report a case of a 59-year-old woman with rectal lymph node metastases in recurrent ovarian carcinoma, who survived for 45 months without rectal obstruction after SDS, whose treatment goal was gradually changed from curative to palliative chemotherapy, and was followed up until autopsy. Other authors have reported the role of ^18^F-FDG PET/CT in the diagnosis of inguinal lymph node metastases from ovarian carcinoma [8,9]. To our knowledge, this is the first report describing the value of ^18^F-FDG PET/CT in detecting rectal lymph node metastasis and in treatment planning for recurrent ovarian carcinoma.

Rectal lymph node metastases may be more frequent in ovarian carcinoma than previously believed [4,5]. Gynecologists require anatomical knowledge of lymph nodes around the rectum, because once ovarian carcinoma infiltrates the rectal wall, the pattern of lymphatic spread may be similar to that of primary rectal carcinoma [4,5]. However, the location, distribution, and size of the lymph nodes around the rectum remain under debate [6,7]. Moreover, there is no consensus on the nomenclature for lymph nodes around the rectum, that is, the terms ‘epirectal’ , ‘pararectal’ , ‘perirectal’ , and ‘mesorectal’ are all used in reports by different researchers [3-7]. Further studies are required to establish more precise topography and a strict nomenclature for lymph nodes around the rectum.

Visual images are essential for the interpretation of recurrent ovarian carcinomas, since an elevated CA125 value does not indicate the size or location of the recurrent tumor. The PET/CT scan has additional value because it combines functional and metabolic characteristics with morphological and anatomical images. Therefore, ^18^F-FDG PET/CT has high sensitivity in detection of metastases in lymph nodes larger than 7 mm [11,12], which proved to be the case here: two mesorectal lymph nodes (12 and 26 mm) and one pararectal lymph node (18 mm) were detected with ^18^F-FDG PET/CT prior to SDS (Figures 3 and 4). Using only conventional CT, it remains challenging to distinguish between metastasis and reactive swelling in individual lymph nodes, to identify the mesorectal lymph node adjacent to the sacral bone (Figure 3: 3A), and to discriminate lymph node metastasis from rectal wall thickness (Figure 3: 3C). In the present case, conventional CT and PET/CT scans had sensitivities of 25% (excluding indeterminate findings) and 75%, respectively. Recent studies have reported that ^18^F-FDG PET/CT has higher patient-based sensitivity, specificity, positive predictive value, and accuracy (53% to 97%, 80% to 97%, 89% to 98%, and 68% to 92%, respectively) than conventional CT for recurrence of ovarian cancer [11]. Thus, ^18^F-FDG PET/CT may be a superior diagnostic tool than conventional CT for detecting rectal lymph node metastasis in recurrent ovarian carcinoma [10-12].

There has not been much focus on preoperative planning using ^18^F-FDG PET/CT in recurrent ovarian carcinoma. Lenhard *et al*. [10] reported that recurrent tumors were completely removed without macroscopic remnants in 21 of 24 patients in whom the possibility of complete cytoreduction had been predicted by PET/CT imaging. Similarly, in the present case, the surgery accomplished macroscopic complete resection as predicted by ^18^F-FDG PET/CT. Later, ^18^F-FDG PET/CT identified multiple metastases in the pelvic lymph nodes (Figure 5); at that time, we gradually changed the treatment goals from curative to palliative chemotherapy. The patient not only survived for a longer time after SDS (45 months) than did other such patients (mean survival time, 32 months) in previous studies [13], but also experienced no rectal obstruction, which improved the patient’s quality of life. Thus, ^18^F-FDG PET/CT could play a determinative role in patient management [10].

Dual-point time (DPT) ^18^F-FDG PET/CT is a semiquantitative technique in which analyses are performed using both early and delayed maximum standardized uptake values (SUV_max_). ^18^F-FDG is not specific for malignancy; for example, activated inflammatory cells have increased expression of glucose transporters and increased ^18^F-FDG uptake. DPT ^18^F-FDG PET/CT is clinically useful, because a lesion is likely to be malignant if SUV_max_ is increasing over time, whereas it is likely to be benign if SUV_max_ is stable or decreasing [14]. Chan *et al*. [15] reported a significant improvement in the sensitivity of delayed SUV_max_ (94% at a mean of 155 min) compared with the sensitivity of early SUV_max_ (77% at a mean of 64 min) in detecting 133 malignant lesions among 53 patients. In the present study, we confirmed the lymph nodes as metastatic based on early (at 60 min) and delayed (at 120 min) SUV_max_ data (Figures 3 and 5). Thus, DTP ^18^F-FDG PET/CT provides a more accurate diagnosis than conventional CT for recurrent ovarian carcinoma.

In 2006, Chi *et al*. [2,16] established a practical guideline for the management of recurrent ovarian carcinoma based on data from 153 patients treated at their institute. They proposed that the disease-free interval and the number of recurrent sites should be used as selection criteria for whether to offer SDS. ^18^F-FDG PET/CT provides more accurate information on the number of recurrent sites compared with conventional CT [10-12]. Thus, ^18^F-FDG PET/CT may become an indispensable imaging modality for determining whether to offer SDS or avoid unnecessary surgery. Further studies are needed to determine whether treatment decisions using ^18^F-FDG PET/CT provide a survival benefit to patients with recurrent ovarian carcinoma.

## Conclusions

^18^F-FDG PET/CT may be valuable for detecting rectal lymph node metastasis, and can play an essential role in planning treatment of recurrent ovarian carcinoma.

## Consent

Written informed consent was obtained from the patient’s daughter for publication of this case report and all accompanying images. A copy of the written consent is available for review by the Editor-in-Chief of this journal.

## Abbreviations

18F-FDG: ^18^F-fluorodeoxyglucose; CA125: Cancer antigen 125; DPT: Dual-point time; PET/CT: Positron emission tomography/computed tomography; SDS: Secondary debulking surgery; SUVmax: Maximum standardized uptake value.

## Competing interests

The authors declare that they have no competing interests.

## Authors’ contributions

KK drafted the manuscript and searched the literature. KK and MT were involved in the treatment of the patient. TO evaluated the ^18^F-FDG PET/CT data for the recurrent tumors. HS and CW reported the pathological findings and prepared the photographs. MO reviewed and edited the manuscript. All authors have read and approved the final manuscript.
